# Alternative Solid Activators from Waste Glass for One-Part Alkali-Activated Fly Ash/Red Mud Cements

**DOI:** 10.3390/ma16206707

**Published:** 2023-10-16

**Authors:** Nicolaie Marin, Cristina Orbeci, Liliana Bobiricǎ, Ileana Rău, Calin Deleanu, Elena Iuliana Bîru, Paul Octavian Stănescu, Andrei Constantin Berbecaru, Ecaterina Matei, Constantin Bobiricǎ

**Affiliations:** 1 Department of Analytical Chemistry and Environmental Engineering, National University of Science and Technology Politehnica Bucharest, 1-7 Gh. Polizu, 011061 Bucharest, Romania; 2 Department of General Chemistry, National University of Science and Technology Politehnica Bucharest, 1-7 Gh. Polizu, 011061 Bucharest, Romania; 3 “C.D. Neniţescu”, Institute of Organic and Supramolecular Chemistry, 202-B Spl. Independenţei, 060023 Bucharest, Romania; 4 Advanced Polymer Materials Group, National University of Science and Technology Politehnica Bucharest, 1-7 Gh. Polizu Street, 011061 Bucharest, Romania; 5 Department of Metallic Materials Processing and Eco-Metallurgy, National University of Science and Technology Politehnica Bucharest, 313 Spl. Independenţei, 060042 Bucharest, Romania

**Keywords:** waste glass, alkali fusion, solid activators, fly ash, red mud, one-part alkali-activated materials

## Abstract

Solid activators based on waste glass for the manufacture of one-part alkali-activated fly ash/red mud materials were synthesized, characterized, and tested in this work. The synthesis was carried out via alkaline fusion with sodium hydroxide at different reaction temperatures and at different sodium hydroxide/waste glass mass ratios. The results showed that the reaction temperature decisively influences the properties of the obtained solid activators. Thus, the best results regarding the water solubility of solid activators were obtained for the synthesis temperature of 600 °C, regardless of the sodium hydroxide/waste glass mass ratio. Also, the use of these assortments of solid activators led to obtaining the best compressive strength of one-part alkali-activated fly ash/red mud materials. The best results were obtained for the solid activator synthesized at a temperature of 600 °C and a sodium hydroxide/glass waste mass ratio of two.

## 1. Introduction

Currently, there is a high interest in developing one-part alkali-activated cements (“just add water”) due to the advantages they have compared with conventional two-part alkali-activated cements (solid precursors plus alkaline solution). Two-part alkali-activated cements involve the activation of solid precursors (part one of the reaction) with a concentrated alkaline solution (part two of the reaction), while one-part alkali-activated cements involve only a dry mixture, namely solid precursors, and solid alkali activators (only one part of the reaction) to which just water is added [1]. The possibility of being used for in situ applications as well as avoiding the operations of handling large volumes of viscous, corrosive, and dangerous solutions have determined that one-part alkali-activated cements are more and more appreciated compared to two-part alkali-activated cements [2]. 

The manufacture of one-part alkali-activated cements requires the use of some solid activators that can provide alkali cations (Na^+^, K^+^) or alkaline earth cations (Ca^2+^, Mg^2+^) to the system, and facilitate the dissolution of the precursor mixes by attacking their Si-O-Si and Al-O-Al bonds [3]. Thus, a wide variety of solid activators (synthetic or based on industrial waste) have been tested for manufacturing one-part alkali-activated cements. Sodium metasilicates (Na_2_SiO_3_, Na_2_SiO_3_·5H_2_O, Na_2_SiO_3_·9H_2_O), sodium/potassium hydroxide (NaOH/KOH), sodium aluminate (NaAlO_2_), calcium/sodium sulfate (CaSO_4_/Na_2_SO_4_), sodium carbonate (Na_2_CO_3_), potassium silicate (K_2_SiO_3_), calcium oxide/calcium hydroxide (CaO/Ca(OH)_2_), reactive magnesium oxide (MgO), sodium bicarbonate (NaHCO_3_), etc., have been tested as synthetic solid activators [4]. However, many of these solid activators have disadvantages, such as high chemical corrosivity, high hygroscopicity, low availability, and often, their manufacture is associated with a high carbon footprint [5]. 

A series of solid activators based on agricultural and industrial waste were also tested, such as red mud (bauxite residue from the alumina refining industry) [6], concrete waste [7], soda residue (from sodium carbonate production) [8], waste glass [9,10], desulphurization dust (from steel production) [11], and vanadium tailings (vanadium extraction from the vanadium-bearing carbonaceous shale) [12], as well as a series of biomass ashes such as maize stalk ash, maize cob ash, olive stone biomass ash, wood biomass ash, and almond-shell biomass ash. Although promising results have been recorded in many cases, many efforts still need to be made to find an optimal way to synthesize solid activators from these wastes that are efficient from a technical/economic point of view. Often, these wastes require a series of mechanical and chemical treatments before they can be used as solid activators or precursors of solid activators [13,14]. Because the characteristics of these wastes can vary a lot, these preliminary treatments must be optimized every time. A good example is waste glass, often used to manufacture alkaline silicates with a much smaller carbon footprint than the classic manufacturing process, which can be of several categories, namely soda–lime glass, lead glass, borosilicate glass, aluminosilicate glass, etc. Because, at least from a compositional point of view, these glasses are different, the conditions or even the manufacturing methods of alkaline silicates are different. It follows from this that for each type of waste, the methods by which it can be brought into the position of being considered as a solid activator that is usable for the manufacture of one-part alkali-activated cements must be studied closely.

Therefore, the objective of this work is to synthesize, characterize, and test a new class of alternative solid alkaline activators based on Cathode Ray Tube (CRT) waste glass. The study of the synthesis parameters of solid activators via the alkaline fusion of CRT glass (glass from both panels and funnels) with sodium hydroxide is also considered. The assortments of solid activators obtained will be tested for the manufacture of one-part alkali-activated fly ash/red mud cements.

## 2. Materials and Methods

### 2.1. Materials

Cathode Ray Tube (CRT) glass waste (particle size from 1 to 10 mm) was provided by a local collection and processing company of waste from electrical and electronic equipment (WEEE). The CRT glass was ground in a ball mill to a particle size of less than 75 μm (powdery state). Sodium hydroxide (98%, granular, analytical grade) was used in the alkali fusion reaction. Fly ash was provided by a local coal-fired thermal power plant and used without any other treatment. Red mud (bauxite residue) was provided by a local alumina refining industry. Before being used, it was dried and ground to a powdery state. The oxide compositions of CRT glass, fly ash, and red mud (XRF analysis on a Philips PW 4025 MiniPal spectrophotometer) are presented in Table 1.

### 2.2. Solid Alkaline Activator Preparation

The CRT powder and NaOH granules were dry-mixed at three different NaOH/CRT mass ratios until homogeneous mixtures were obtained. The CRT/NaOH mixtures were kept for 2 h in an electric oven at temperatures of 500, 600, and 700 °C (alkali fusion). At the end of the alkali fusion, the synthesized solid alkaline activators were cooled and finely ground. The nine resulting solid alkaline activator assortments are coded and presented in Table 2.

### 2.3. One-Part Alkali-Activated Fly Ash/Red Mud Cements Preparation

The one-part alkali-activated cements were prepared by mechanically dry-mixing fly ash, red mud, and a solid activator until a homogeneous mixture was obtained. The mass ratio of the solid activator/fly ash/red mud is 0.2. The mixture proportions of the one-part alkali-activated cements are presented in Table 3. To better observe the possible differences that appear in terms of cement performances due to the different assortments of solid activators, a single composition of these cements was adopted. A proportion of red mud of 5% (wt.) was established based on previous research on the influence of its proportion in synthesis mixtures on the performance of alkaline-activated materials [15]. Later, the nine types of cement were used for the preparation of one-part alkali-activated fly ash/red mud pastes, which, after 28 days of hardening, were tested for compression strength and subjected to morphological and spectroscopic analysis. For this, the one-part alkali-activated fly ash/red mud cement was mechanically mixed with water at a water/cement mass ratio of 0.5 until a homogenous mixture was obtained. The obtained pastes were cast in cylindrical polypropylene formworks with the dimensions 5 cm (d) × 10 cm (h), and vibrated for 2 min. Next, the formworks were sealed into plastic bags (to avoid carbonation) and cured at 80 °C for 28 days (1 day molded and 27 days demolded).

### 2.4. Characterization and Testing of Solid Alkaline Activators and One-Part Alkali-Activated Fly Ash/Red Mud Pastes

The finely ground solid alkaline activators were tested for their solubility in water, and their contents of soluble Si, and Na was also determined. For this, 1 g of the solid alkaline activator was weighed with analytical precision and contacted with 100 mL of pure water for 24 h under continuous stirring. After that, the suspension was filtered through 0.45 μm pore-size polypropylene membranes. The filters were dried in an electrical oven to a constant mass and weighed with analytical precision. Based on the recorded mass difference, the water solubility of the solid alkaline activators was calculated. The concentrations of sodium and silica were measured in the obtained filtrates. The sodium concentration was measured via flame photometry following the standard method ASTM D 1428-56 T [16], and using a Classic Flame Photometer Model 410 from Sherwood Scientific Ltd. (Cambridge, UK). The silica concentration was measured via spectrometry following the standard method ASTM D 859-00 [17], and using a UV-VIS spectrophotometer Mosel UV-1900 Model from Shimadzu USA Manufacturing, Inc. (Austin, TX, USA).

The nine assortments of solid alkaline activators were also characterized via spectroscopic (FT-IR, XPS, and MAS-NMR) and mineralogical (XRD) analysis. The Fourier transform infrared spectroscopy (FT-IR) analyses were performed on the Bruker VERTEX 70 equipment, employing 32 scans in the 500–4000 cm^−1^ range, with a resolution of 4 cm^−1^ in total attenuated reflection mode (ATR) by employing a Ge crystal. The X-ray photoelectron spectroscopy (XPS) analyses were conducted using Thermo Scientific K-Alpha equipment (Thermo Fisher Scientific, Waltham, MA, USA), using a monochromatic Al Kα source (1486.6 eV), at a pressure of 2 × 10^−9^ mbar. The binding energy was calibrated by placing the C 1s peak at 284.8 eV as the internal standard. The ^29^Si Magic Angle Spinning–Nuclear Magnetic Resonance (^29^Si MAS-NMR) spectra were recorded on a Bruker AVANCE Neo 400 spectrometer (9.4 Tesla, 4 mm ZrO2 rotors, ambient temperature, spinning rate 10 kHz). The ^29^Si spectra were obtained at a 0.04 s acquisition time, with a 2 s relaxation delay, and with several scans ranging between 8192 and 20,480 scans, depending on the sample. The resonance frequency was 79.5 MHz. The chemical shifts were electronically referenced relative to tetramethylsilane (TMS) as the external standard. The X-ray diffractometry (XRD) was performed on a Panalytical X’Pert Pro MPD (Multipurpose Difractometer). Data collection was performed over a range from 10 to 90° with a scanning rate of 1.5° (2θ)/min with CuKα radiation (45 kV, 40 mA, λ = 1.5406 nm). The crystal phases were identified by referencing diffraction patterns in a licensed library from the International Centre for Diffraction Data (ICDD).

The one-part alkali-activated fly ash/red mud pastes were characterized from a morphological and spectroscopic point of view (SEM-EDX and FT-IR). They were also tested for compressive strength. Scanning electron microscopy with energy dispersive X-ray (SEM/EDX) spectroscopy analyses were performed on a Quanta 650 FEG scanning electron microscope (SEM) equipped with an EDX analyzer, operated at 10 kV. The compressive strength tests were carried out according to standard method ASTM C39/C39M-14 [18] for cylindrical specimens on an Instron 3382 instrument equipped with a 100 kN load cell at room temperature, at a 1 mm/min compression rate.

## 3. Results and Discussion

### 3.1. Physico-Chemical Characterization of Solid Activators

#### 3.1.1. Water Solubility

**Figure 1:**
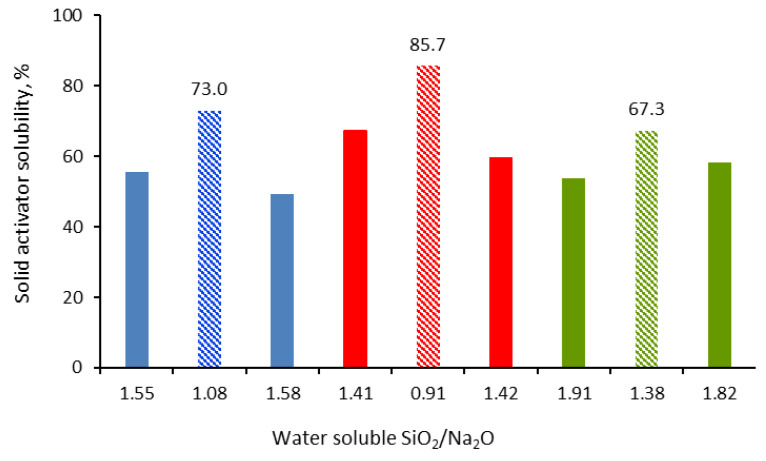
Water solubility of the synthesized solid activators.

**Figure 2:**
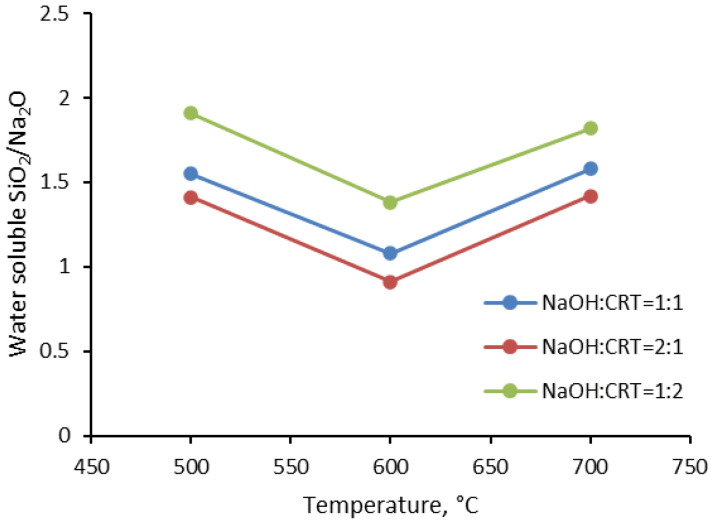
Water soluble SiO_2_/Na_2_O weight ratio (WR) as a function of alkali fusion temperature.

The water solubility of the obtained solid activators is their defining characteristic in manufacturing one-part (“just add water”) alkali-activated cements. The solid activators must provide the system with the necessary alkalis to attack the T-O-T bonds (T: Si or Al) in the precursors with the formation of T-OH groups that subsequently condense and form aluminosilicate gels. Therefore, the first condition for a solid activator to be effective is that it is easily soluble in water [19]. As can be seen from Figure 1, the solubility of the assortments of solid activators depends on the synthesis conditions. It seems that the assortments with a NaOH/CRT ratio of two, synthesized at a temperature of 600 °C, have the highest solubility. At the same time, this corresponds to the lowest SiO_2_/Na_2_O water soluble mass ratio, which, in turn, depends on the synthesis temperature of the solid activators (Figure 2). The synthesis temperature of solid activators controls the type of sodium silicates formed and their weight in the composition of the activator. As shown in the XRD analysis, the assortment of solid activators that were synthesized at a temperature of 600 °C mainly contain sodium metasilicate (Na_2_SiO_3_) with highly soluble silica in water [20]. 

Regarding the variation of the NaOH/CRT mass ratio, the results clearly indicate that increasing the amount of sodium hydroxide leads to an increase in the percentage of the solubilized activator (Figure 1). Considering the amounts of CRT used in the reaction, as well as the initial composition of the CRT in terms of SiO_2_, its reacted and water-dissolved percentage for all three NaOH/CRT mass ratios was calculated. In this respect, the SiO_2_ mass percentage varies slightly in the ranges of 67–69% for the temperature of 500 °C, 91–92% for 600 °C, and 79–81% for 700 °C. Similar results have been reported in the literature [21]. It can be clearly seen that significant differences are recorded for different reaction temperatures, which are therefore the main operating parameter of the synthesis. The temperature of the alkali fusion reaction plays an important role in terms of the phase change of the reactants (initially introduced in the solid phase) and therefore the homogeneity of the reaction mixture. The minimum temperature of 500 °C was chosen to be above the melting temperature of sodium hydroxide (318 °C), and at the limit of the glass transition temperature of CRT glass. As reported in the literature, this varies in the range of 462–523 °C for this type of glass [22]. Since the exact value was not measured for the CRT glass used in this work, it is very possible that it is higher than 500 °C. Therefore, the lower solubility in water of the solid activators synthesized at 500 °C, compared to that at 600 °C, could be attributed to a poor reaction between reactants (initially introduced in the solid phase), probably induced by the fact that the synthesis temperature could have been below the glass transition temperature of the CRT glass that was used. At higher reaction temperatures, more complex forms of sodium silicates (i.e., Na_2_Si_2_O_5_) with lower solubility in water usually appear [23,24]. This fact was also shown in the XRD analyses in this paper (figure in Section 3.1.5) and could be the cause of the lower solubility in water of the solid activators synthesized at 700 °C, compared to those synthesized at 600 °C.

It should also be mentioned that, most likely, not all the sodium hydroxide that is used reacts with SiO_2_, with part of it carbonating in the furnace atmosphere, as was highlighted in the XRD and FTIR analyses, or it reacts with other glass components. The formation of sodium carbonate could be beneficial to the system because it itself can act as an activator [25,26,27]. The lack of NaOH peaks in the XRD patterns may be due to the decomposition of sodium hydroxide to sodium oxide at the temperatures investigated. If the amount of soluble silica remains approximately constant regardless of the NaOH/CRT ratio, then the difference in the total solubility of the solid activator at different NaOH/CRT ratios can be mostly attributed to the solubilization of the unreacted sodium hydroxide and the sodium carbonate formed, which are in different amounts in the initial reaction mixtures. All that which is insoluble in water in the composition of solid activators can act as fillers in the manufacture of one-part alkali-activated materials. In the following, the discussion will be focused only on the NaOH/CRT 2:1 assortments, obtained at different alkaline fusion temperatures, for which the best results in terms of water solubility were obtained.

#### 3.1.2. MAS-NMR Spectroscopy

**Figure 3:**
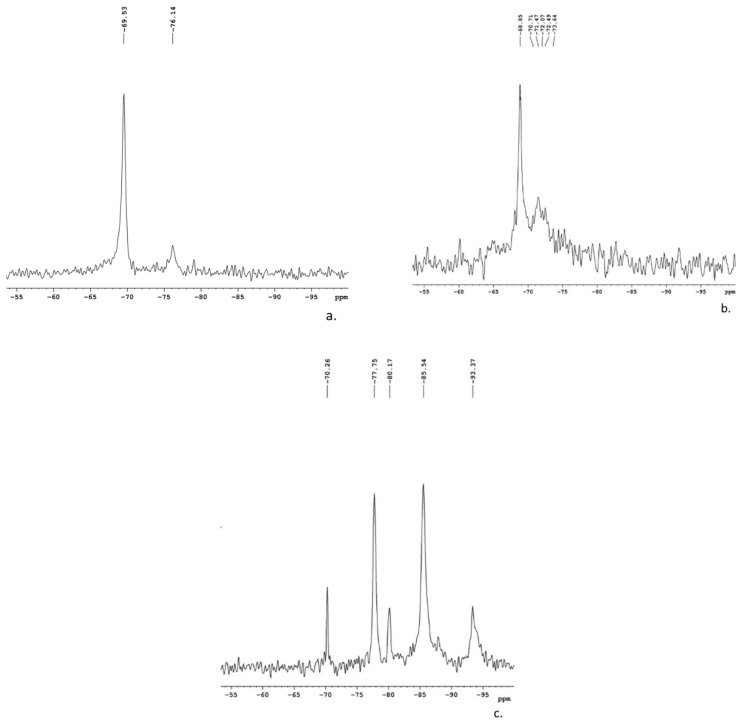
^29^Si MAS-NMR spectra of the solid activators: (**a**) NaOH/CRT = 2:1 600; (**b**) NaOH/CRT = 2:1 500; (**c**) NaOH/CRT = 2:1 700.

The chemical environments of the Si atoms in the solid activators that were synthetized at different alkali fusion temperatures were determined based on ^29^Si MAS-NMR measurements. As can be seen in Figure 3, the degree of connectivity of the SiO_4_ tetrahedra in the solid activators is influenced by the synthesis temperature. Thus, for the assortment of NaOH/CRT = 2:1 600, the peaks appearing in the spectrum at −68.85 ppm, −70.71 ppm, and −73.64 ppm are associated with Q_1_ units [28]. For the NaOH/CRT = 2:1 500 assortment, the peaks appearing in the spectrum at −69.53 ppm and −70.71 ppm are associated with the units Q_1_ and Q_2_, respectively. For the NaOH/CRT = 2:1 700 assortment, the transition to Q_2_ units (−77.75 ppm, −80.17 ppm) and Q_3_ units (−85.59 ppm, −93.71 ppm) is evident [29,30]. It is known that as the proportion of Q_3_ and Q_4_ units increases, the degree of connectivity of the SiO_4_ tetrahedra increases, and therefore their reactivity in the activation process decreases [31].

#### 3.1.3. X-ray Photoelectron Spectroscopy

##### O 1s Spectra

**Figure 4:**
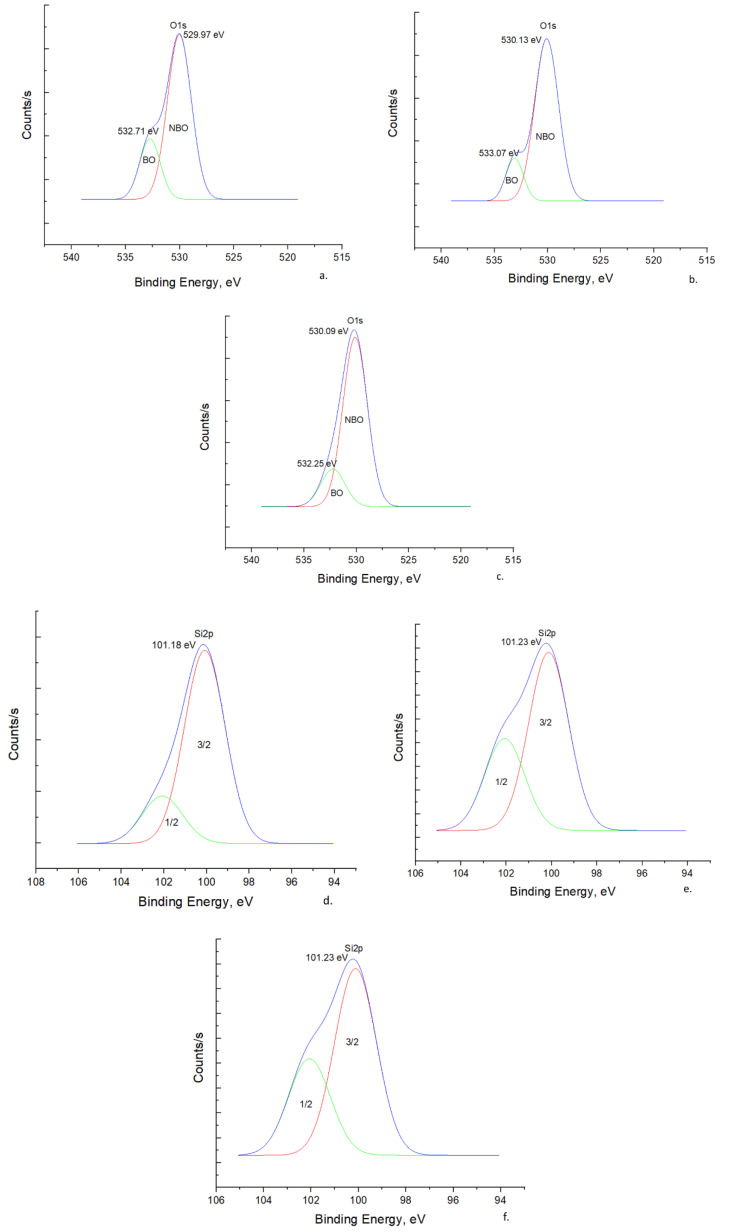

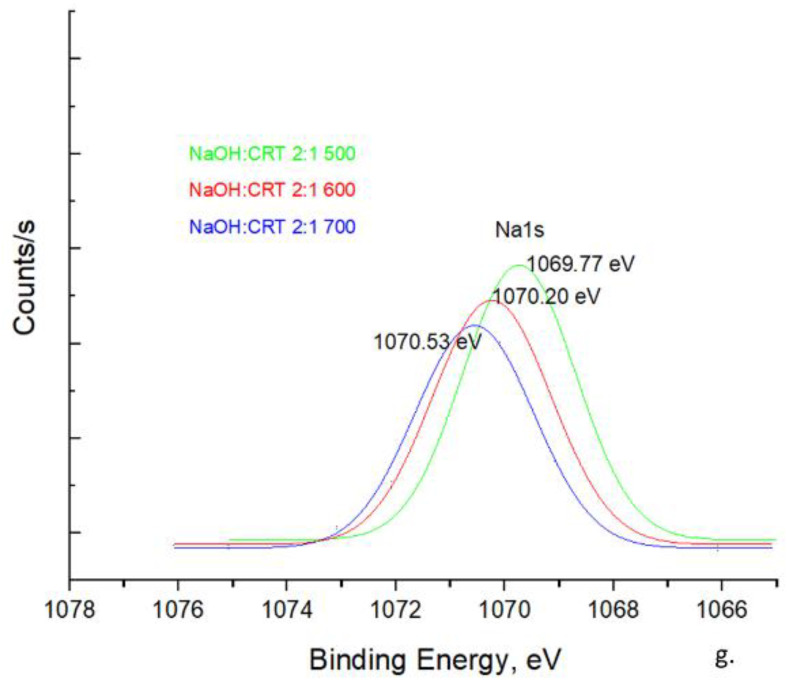
XPS spectra with Gaussian peak fittings: (**a**) O 1s for NaOH/CRT 2:1 500; (**b**) O 1s for NaOH/CRT 2:1 600; (**c**) O 1s for NaOH/CRT 2:1 700; (**d**) Si 2p for NaOH/CRT 2:1 500; (**e**) Si 2p for NaOH/CRT 2:1 600; (**f**) Si 2p for NaOH/CRT 2:1 700; (**g**) Na 1s.

In the alkaline fusion reaction of CRT glass with NaOH, the introduced Na_2_O breaks the tetrahedral network of SiO_4_ and, at the same time, introduces a new oxygen atom into the system. In this situation, not all oxygen atoms will be linked to two silicon atoms each (bridging oxygen, BO: Si-O-Si), but there will also be some that will be linked to one silicon atom each (non-bridging oxygen, NBO: Si-O^−^), thus acquiring a negative charge. Next, the sodium ions associate with the negatively charged non-bridging oxygen atoms (Si-O^−^Na^+^) [32]. Since BO has a poor electron density, a larger amount of energy is required to eject a photoelectron from the O 1s orbital and therefore, a larger binding energy will be recorded. On the contrary, NBO has a higher electron density than BO; thus, a smaller amount of energy is required to reject a photoelectron from the O 1s orbital and, therefore, a lower binding energy will be recorded [33]. The measured XPS O 1s spectra with Gaussian peak fittings are presented in Figure 4a–c. Based on the above, the peaks with a higher binding energy are associated with the BO contribution (green curves), while the peaks with a lower binding energy are associated with the NBO contribution (red curves). The peaks were fitted freely using a least squares best fit criterion. As can be seen, there is a tendency to shift the bonding energy for both BO and NBO towards higher values for the solid activator synthesized at 600 °C (Figure 4b), compared to the one synthesized at 500 °C (Figure 4a). However, this trend is not maintained for the activator obtained at 700 °C (Figure 4c), with the bond energy being shifted again towards lower values. This behavior could be explained based on changes in the electron density of oxygen atoms as a function of the number of silicon atoms to which they are linked. In this respect, the increase or decrease in the number of silicon atoms leads to the decrease or increase in the electron density of all the oxygen atoms to which they are linked, causing a shift in the binding energy for both BO and NBO towards higher or lower values, respectively [34]. If this hypothesis is analyzed based on the soluble SiO_2_/Na_2_O ratio of the solid activators measured in this work from the water solubility tests (Figure 1), a good agreement can be found between the values of this ratio and the results from the O 1s spectra. In this respect, the decrease in the soluble SiO_2_/Na_2_O ratio from 1.08 for the solid activator assortment that was synthesized at 500 °C to 0.91 for the assortment that was synthesized at 600 °C reflects more silicon atoms in the system. This could lead to a decrease in the electron density of oxygen atoms, and thus the binding energy for both BO and NBO is shifted to higher values. Similarly, the increase in the SiO_2_/Na_2_O ratio from 0.91 for the solid activator assortment that was synthesized at 600 °C to 1.82 for the assortment that was synthesized at 700 °C reflects a decrease in the number of Si atoms in the system. This could also increase the electron density of the oxygen atoms and thus shift the binding energy for both BO and NBO to lower values. It also seems that the presence of a larger amount of soluble SiO_2_ in the system leads to an increase in the NBO fraction at the expense of the BO fraction. Thus, following the integration of the surfaces under the corresponding curves for BO and NBO, it was established in the case of the assortment synthesized at 500 °C that the fraction of BO is 0.27 and that of NBO 0.73; for the assortment synthesized at 600°, the fraction of BO is 0.23 and that of NBO is 0.77; and for the assortment synthesized at 700 °C, the fraction of BO is 0.25 and that of NBO is 0.75. 

##### Si 2p Spectra

The measured XPS Si 2p spectra with Gaussian peak fittings are presented in Figure 4d–f. The binding energy of the Si 2p peak varies similarly to the variation of the O 1s peak. Thus, the binding energy is shifted to higher values for the mixture synthesized at 600 °C (Figure 4e) compared to the one synthesized at 500 °C (Figure 4d), and then later it is shifted again to lower values in the case of the mixture synthesized at 700 °C (Figure 4f). Increasing the Na_2_O content in the system leads to an increase in the electron density on the Si nuclei, which has the effect of shifting the binding energy of Si 2p_3/2_ to lower values. Since the electronegativity of Na is much lower than that of Si, the electron density over the NBO atoms should be higher than that over the BO atoms, which are bound only to Si, and this should increase with the increase in the number of Na atoms in the system. Therefore, as the number of NBO atoms bound to the central Si atom is higher, the electron density above the central Si atom is higher, and therefore the binding energy of the Si 2p peak will be lower. Thus, it becomes obvious that the degree of connectivity of the SiO_4_ tetrahedron (Q_n_) depends on the number of NBO atoms linked to the central Si atom, and the binding energy of Si 2p systematically decreases from Q_4_ units to Q_0_ units [24]. These results are in good agreement with those obtained from the ^29^Si MAS-NMR analysis (Figure 3), with the assortment of solid activators synthesized at 600 °C being characterized by Q_1_ units, compared to the other assortments, which seem to be characterized mainly by Q_2_-Q_3_ units.

##### Na 1s Spectra

The measured XPS Na 1s spectra with Gaussian peak fittings are presented in Figure 4g. As can be seen, although there is a small shift in the bond energy with the increase in the synthesis temperature of the solid activators, it can still be stated that Na exists in a one-bond configuration.

#### 3.1.4. FT-IR Spectroscopy 

**Figure 5:**
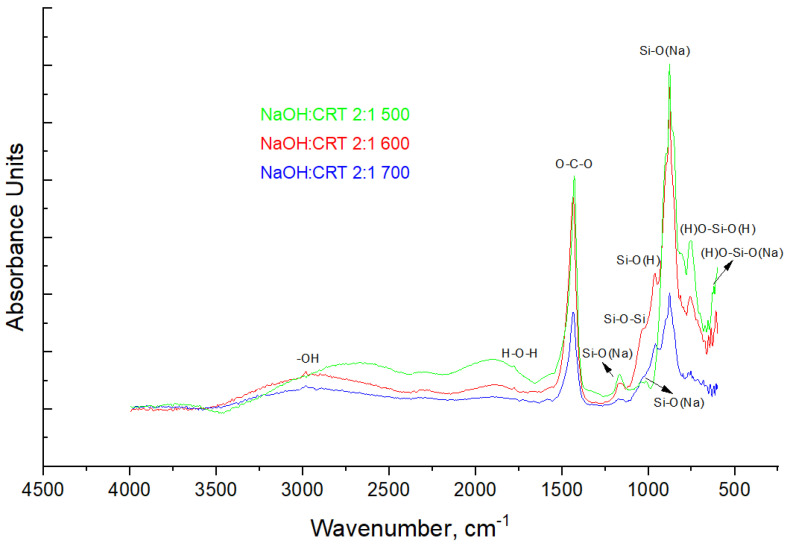
FT-IR spectra of the solid activators.

The FT-IR spectra of the solid activators synthesized at different reaction temperatures (Figure 5) highlight a main sharp absorption band at 877.54 cm^−1^, assigned to the symmetric stretching vibration of the Si-O(Na) bonds. The absorption bands located in the spectra at 621.03 cm^−1^ and 754.11 cm^−1^ are assigned to the asymmetric deformation vibration of (H)O-Si-O(Na) bonds and the asymmetric deformation vibration of (H)O-Si-O(H) bonds, respectively [35]. The sharp shoulder appearing at the wavelength of 960.48 cm^−1^ could be assigned to the Si-O(H) bonds, and the broad shoulder appearing at the wavelength of 1026.05 cm^−1^ is attributed to the asymmetric stretching vibration of the Si-O-Si bonds [15]. The absorption band at 1161.06 cm^−1^ is attributed to the asymmetric stretching vibration of the Si-O(Na) bonds. The sharp absorption band that appears in the spectra at the wavelength of 1427.21 cm^−1^ is assigned to the O-C-O stretching vibration bonds in Na_2_CO_3_, formed due to the carbonation of NaOH during the alkali fusion [36]. The absorption bands that appeared at wavelengths of 1774.38 cm^−1^ and 2983.65 cm^−1^ are assigned to the bending vibration of the H-O-H molecules (hydrogen bonding) and the stretching vibration of O-H (free OH group). 

It should be mentioned that the results obtained in the FT-IR analysis are consistent with those obtained in the MAS-NMR and XPS analyses. Thus, the absorption band that appears at approximately 880 cm^−1^ could be assigned to Q_1_ units (Si-O-3NBO), and the one that appears at approximately 958 cm^−1^ could be assigned to Q_2_ units (Si-O-2NBO). Also, the broad shoulder that appears in the range of 1020–1040 cm^−1^ can include two overlapping peaks that can be attributed to the Si-O-Si siloxane bond and Q_3_ units (Si-O-1NBO) [24,37].

#### 3.1.5. X-ray Diffractometry

**Figure 6:**
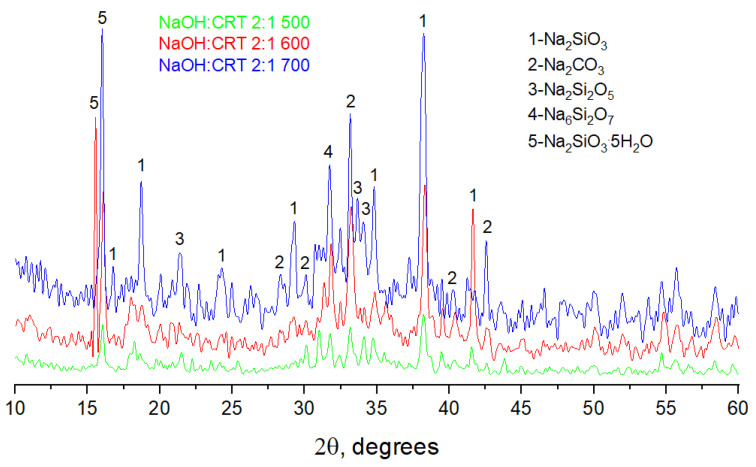
XRD patterns of the solid activators.

The XRD diffraction patterns of the solid activators (Figure 6) highlight some low and scattered bands, attributed to a low-order crystalline structure that seems to be specific to the formation of a Si-rich gel. In addition, in the 30–35 2θ interval, a broad band can be observed, this most likely being associated with unreacted glass or with the formation of silica-rich polymers [27]. Sharp bands associated with the crystalline phases of activators such as sodium metasilicate (Na_2_SiO_3_), sodium silicate pentahydrate (Na_2_SiO_3_·5H_2_O), and sodium carbonate (Na_2_CO_3_) are present in all three spectra [38]. In the spectra of the solid activators synthesized at reaction temperatures of 600 °C and 700 °C, new sharp bands appear, associated with other species of sodium silicates such as sodium disilicate (Na_2_Si_2_O_5_) and sodium pyrosilicate (Na_6_Si_2_O_7_). These results are in agreement with those obtained in the NMR, XPS, and FTIR analyses, and demonstrate the fact that, as the reaction temperature increases, more complex species of sodium silicates are formed, such as Na_2_Si_2_O_5_ (i.e., Na_2_O·2SiO_2_) and Na_6_Si_2_O_7_ (i.e., 3Na_2_O·2SiO_2_) [24].

### 3.2. Mechanical and Microstructural Characterization of One-Part Alkali-Activated Materials

#### 3.2.1. Compressive Strength

**Figure 7:**
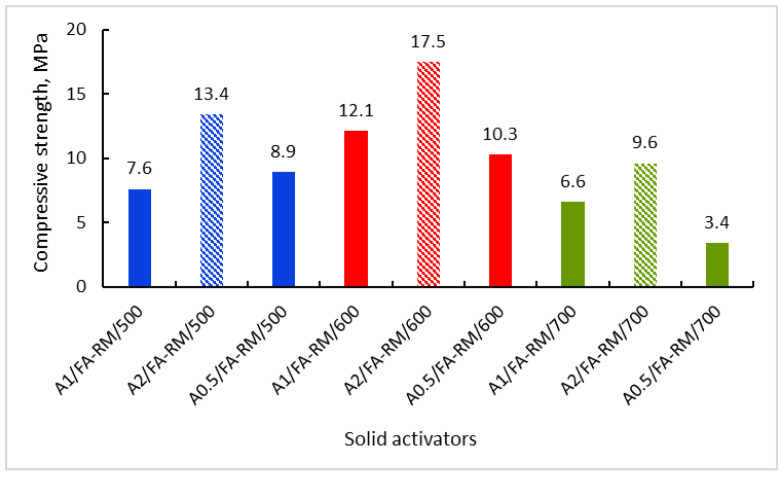
Compressive strength of the one-part alkali-activated materials.

Figure 7 shows the compressive strength of the one-part alkali-activated materials, manufactured with the solid activators synthesized in this work. As can be seen, the results regarding the compressive strength are in close agreement with the results regarding the solubility of the solid activators in water (Figure 1). Thus, the materials that were manufactured with assortments of solid activators, synthesized at NaOH/CRT mass ratios of two and reaction temperatures of 600 °C, show the best compressive strength. Therefore, the degree of solubility in water of the solid activators is their defining property, on which the properties of alkaline-activated materials later depend. Again, the synthesis temperature of 600 °C gives the best results, regardless of the initial mass ratio of the reactants. If the evolution of the compressive strength is analyzed for the activator assortments that were synthesized at a mass ratio between the reactants of two, and at different synthesis temperatures, the result is that the compressive strength of the A2/FA-RM/600 assortment is 1.3 times higher than that of the A2/FA-RM/500 assortment and 1.8 times higher than that of the A2/FA-RM/700 assortment. However, according to the ASTM C90-16a standard [39], only the A2/FA-RM/500 and A2/FA-RM/600 assortments have a compressive strength higher than 12.4 MPa, which recommends them for the manufacture of load-bearing concrete masonry units. Moreover, the TCLP (toxicity characteristic leaching procedure) compliance leaching tests showed a very low degree of leaching of the contaminants (initially present in the secondary raw materials used) in these one-part alkali-activated assortments [40]. Thus, the concentration of contaminants in the leaching solution is approximately 30 times lower than that imposed by the leaching standard. This is another aspect that strengthens the belief that these types of one-part alkali-activated assortments can be used in the field of construction. In the following, only these three types of one-part alkali-activated materials will be characterized.

#### 3.2.2. SEM-EDX Spectroscopy

**Figure 8:**
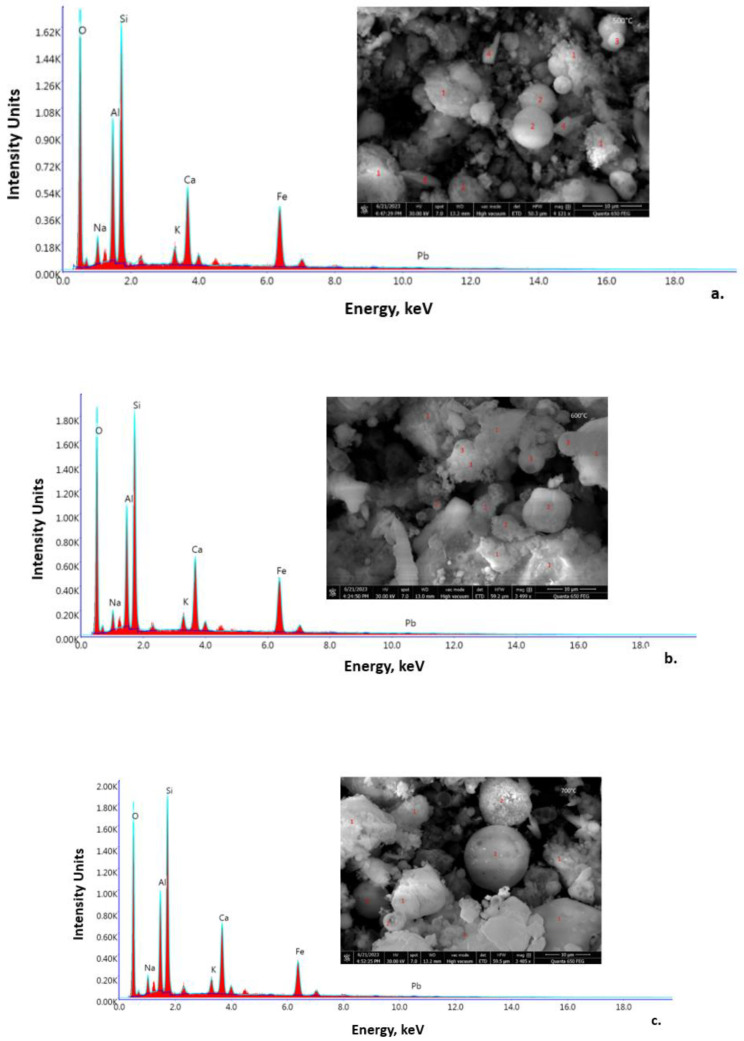
SEM-EDX of the one-part alkali-activated materials: (**a**) A2/FA-RM/500; (**b**) A2/FA-RM/600; (**c**) A2/FA-RM/700.

The SEM micrographs and EDX spectra of the one-part alkali-activated materials are shown in Figure 8. The micrograph of assortment A2/FA-RM/500 (Figure 8a) highlights areas with amorphous material (points 1) that are specific to aluminosilicate gels formed following the alkaline activation process. A series of spherical particles that are specific to fly ash, which are covered (points 3) or partially covered (points 2) with amorphous gel, can also be observed. These can be considered as partially reacted particles that have not completely adhered to the amorphous mass of the alkaline-activated material. There are also individual groups of particles with irregular shapes (points 4) that most likely come from the red mud or from unreacted CRT glass, and that did not participate in the alkaline activation process; they only play the role of filler. The micrograph corresponding to assortment A2/FA-RM/600 (Figure 8b) highlights a much more extensive mass of aluminosilicate amorphous material (points 1) than the micrograph corresponding to assortment A2/FA-RM/500 (Figure 8a) and the one corresponding to assortment A2/FA-RM/700 (Figure 8c). Also, it seems that the proportion of fly ash particles that are completely (points 2) or incompletely (points 3) covered with amorphous gel is much lower in the A2/FA-RM/600 assortment compared to the assortments A2/FA-RM/500 and A2/FA-RM/700. No other isolated formations are observed, a sign that they are completely embedded in the predominant amorphous structure of the material. Regarding assortment A2/FA-RM/700, its micrograph (Figure 8c) highlights an amorphous mass (points 1) that is much less compact, and it even has a slightly different structure than that of the assortments A2/FA-RM/500 and A2/FA-RM/600, with some amorphous areas even being isolated from other formations in the activated mixture. Completely unreacted fly ash particles can also be seen (points 3), and those covered with amorphous material (points 2) are isolated from the compact mass of the reacted material. 

The EDX analysis indicates slight changes in the composition of the amorphous gel phases depending on the type of solid activator used. Thus, the results indicate Si/Al and Na/Al molar ratios of 1.65 and 0.30 for A2/FA-RM/500, 1.52 and 0.86 for A2/FA-RM/600, and 1.72 and 0.48 for A2/FA-RM/700. Although all the three assortments have a value of the Si/Al molar ratio in the range of 1–3, which is specific to N-A-S-H gel sodium aluminosilicate hydrate, the assortment A2/FA-RM/600 has a composition that suggests a better-developed aluminosilicate network; in this case, the Na/Al molar ratio is close to unity. Since, for charge balance in the bonding network, the optimum theoretical Na/Si molar ratio required is 1.00 [41], the assortment A2/FA-RM/600 comes closest to this value (0.57) compared to the other two (0.18 for A2/FA-RM/500 and 0.28 for A2/FA-RM/700). Regarding the Ca/Si molar ratio, its value is around 0.4 for all three types of activators. This could indicate the formation of a low calcium C–S–H (calcium silicate hydrate) gel, C-A-S-H (calcium aluminosilicate hydrate) or a Ca-substituted N-A-S-H gel ((N, C)–A–S–H) [42].

#### 3.2.3. FT-IR Spectroscopy

**Figure 9:**
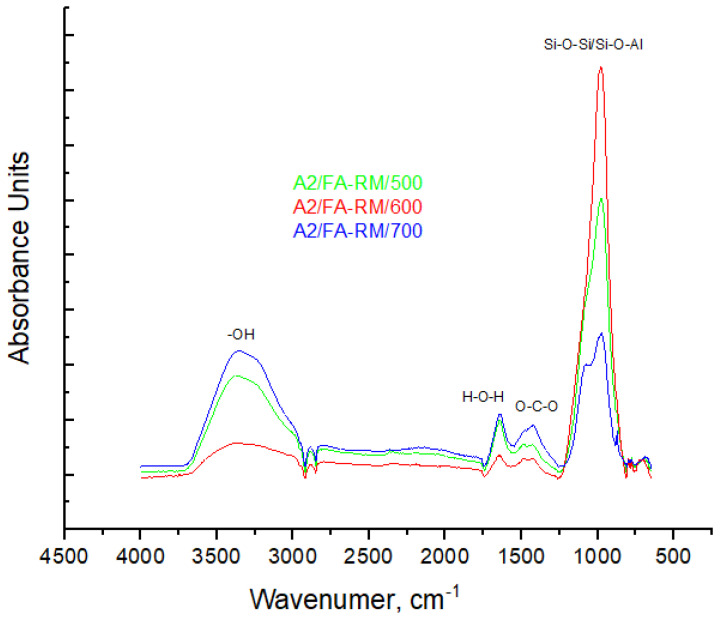
FT-IR of the one-part alkali-activated materials.

The FT-IR spectra (Figure 9) highlights that the main absorption band, regardless of the solid activator assortment, falls in the range of 973–980 cm^−1^, which is assigned to the asymmetric stretching vibration of Si–O–Si or Si–O–Al bonds in aluminosilicates phases [43]. There is a shift in the Si–O–Si/Si–O–Al bond toward a higher wavenumber, namely from 973 cm^−1^ for the A2/FA-RM/500 assortment to 980 cm^−1^ for the A2/FA-RM/600 assortment, followed by a shift in this bound to lower wavenumber, namely to 975 cm^−1^ for A2/FA-RM/700. Shifting this bound toward a higher wavenumber indicates a higher polymerization degree of the aluminosilicate phases in the one-part alkali-activated materials, and vice versa [44]. Also, the increase in the intensity of the peak corresponding to this bond for assortment A2/FA-RM/600 indicates a higher degree of polymerization compared to the other two assortments. This result is in good agreement with the results obtained in the SEM-EDX analysis, and it is also reflected in the results regarding the compressive strength (i.e., as the compressive strength is higher, the higher the degree of polymerization of the aluminosilicate phases). The absorption bands appearing at the wavenumber in the range of 1422–1431 cm^−1^ is assigned to the O–C–O stretching vibration of carbonate groups [45]. The absorption bands appearing at the wavenumber of around 1645 cm^−1^ is assigned to the bending vibration of H–O–H bonds (interlayer-adsorbed water within the activated mixtures), and the band appearing at around 3365 cm^−1^ is assigned to the stretching vibration of O-H (free OH group). The significant difference regarding the intensity of the peak corresponding to the free OH group of assortment A2/FA-RM/600 compared to assortments A2/FA-RM/500 and A2/FA-RM/700 could indicate a greater weight of solid sodium hydroxide in the last two [46], and implicitly, a low degree of its consumption in the reaction of alkaline fusion, by which the solid activators were synthesized.

## 4. Conclusions

The objective of this work was to synthesize, characterize, and test a new class of alternative solid alkaline activators based on CRT waste glass. The synthesis of the solid activators was carried out via alkaline fusion with sodium hydroxide, and their testing was carried out by manufacturing one-part alkali-activated cements based on fly ash and red mud. The results regarding the synthesis of the solid activators showed that the reaction temperature plays an important role in terms of their composition and characteristics. As proven through spectroscopic and mineralogical analyses, the assortments of solid activators that were synthesized at a temperature of 600 °C have a metasilicate-like composition, which gives them a better solubility in water compared to the other assortments of solid activators synthesized at other reaction temperatures. It seems that, at the synthesis temperature of 500 °C, the CRT glass is below the glass transition temperature, with the reaction probably being incomplete due to the lack of homogeneity of the reaction mixture, while at the synthesis temperature of 700 °C, much more complex silicate species are formed, which give a high degree of polymerization to the solid activators and, therefore, poor solubility in water.

The morphological and spectroscopic analyses, as well as the compressive strength tests, showed that one-part alkali-activated materials manufactured with solid activators, synthesized at the reaction temperature of 600 °C, have superior characteristics to the other assortments of solid activators. The assortment synthesized at this reaction temperature and at a NaOH/CRT mass ratio of 2:1 (A2/FA-RM/600) presents the most compact aluminosilicate amorphous structure, which gives it the best compressive strength. Therefore, the assortments A2/FA-RM/500 and A2/FA-RM/600 meet the requirements regarding the compressive strength established by the ASTM standard to be used for the manufacture of load-bearing concrete masonry units.

## Figures and Tables

**Table 1:** Oxide composition of the raw materials.

Oxide, (wt. %)	Waste Glass	Fly Ash	Red Mud
SiO_2_	49.60	42.80	11.00
Al_2_O_3_	5.70	21.41	21.00
CaO	1.50	2.21	5.63
Fe_2_O_3_	0.15	9.85	42.78
SO_3_	7.60	2.70	1.10
K_2_O	6.15	2.14	0.37
Na_2_O	5.00	4.13	7.25
P_2_O_5_	0.20	-	4.75
Cl	4.5	3.6	1.40
PbO	12.10	-	-
BaO	6.97	-	-
NiO	0.02	-	-
CuO	0.04	0.02	-
ZnO	0.12	0.03	-
SrO	0.35	-	-
MgO	-	3.30	-
MnO	-	1.08	-
TiO_2_	-	0.87	1.37
Cr_2_O_3_	-	0.03	0.25
Br	-	0.31	-
LOI (loss on ignition)	-	5.52	3.10

**Table 2:** Solid activator assortments’ composition.

Assortments	Alkali Fusion Temperature, (°C)	NaOH/CRT, (wt. Ratio)
NaOH/CRT 1:2 500	500	1:2
NaOH/CRT 1:1 500		1:1
NaOH/CRT 2:1 500		2:1
NaOH/CRT 1:2 600	600	1:2
NaOH/CRT 1:1 600		1:1
NaOH/CRT 2:1 600		2:1
NaOH/CRT 1:2 700	700	1:2
NaOH/CRT 1:1 700		1:1
NaOH/CRT 2:1 700		2:1

**Table 3:** Mixture proportions of one-part alkali-activated cements.

Assortments	Activator Synthesis Temperature, (°C)	Fly Ash, (wt. %)	Red Mud, (wt. %)	Solid Activator, (wt. %)
A0.5/FA-RM/500	500	85	5	10
A1/FA-RM/500				
A2/FA-RM/500				
A0.5/FA-RM/600	600			
A1/FA-RM/600				
A2/FA-RM/600				
A0.5/FA-RM/700	700			
A1/FA-RM/700				
A2/FA-RM/700				

## Data Availability

The data presented in this study are available on request from the corresponding author.
